# Painful Limb Ulcers: A Case Report on Ulcerative Discoid Lupus Erythematosus

**DOI:** 10.7759/cureus.52960

**Published:** 2024-01-25

**Authors:** Shreya N Gupta, Bhushan Madke, Shivani D Jangid, Drishti M Bhatt, Arshiya Khan

**Affiliations:** 1 Dermatology, Venereology and Leprosy, Jawaharlal Nehru Medical College, Datta Meghe Institute of Higher Education & Research, Wardha, IND; 2 Dermatology, Venereology and Leprosy, Jawaharlal Nehru Medical College, Datta Meghe Institute of Higher Education & Research, wardha, IND

**Keywords:** pyoderma gangrenous, connective tissue disorder, anti-nuclear antibodies, scarring disorders, discoid lupus erythematosus

## Abstract

Lupus erythematosus is an autoimmune disorder with varied clinical features. Discoid Lupus Erythematosus (DLE) presents as erythematous, raised plaques. The patients might present with photosensitivity, arthralgia, and nail changes. However, dermoscopy, clinical features, and laboratory markers like high titers of Antinuclear antibodies (ANA) help in clenching the diagnosis. We report a patient in her mid-60s presented with non-healing ulcers oozing pus discharge associated with pain and joint stiffness. Thus, a series of investigations, treatment modifications, and the healing progression of the lesions highlight the importance of retrospective diagnosis.

## Introduction

Cutaneous lupus erythematosus is a multisystem autoimmune disorder predominantly affecting the skin. It presents in various forms, with acute cutaneous lupus, subacute cutaneous lupus, and discoid lupus being the most common [1]. Among these, Discoid Lupus Erythematosus (DLE) is a prevailing subtype, characterized by chronic autoimmune dermatosis. It exhibits features such as scarring and photosensitivity in sun-exposed areas including the nose, forehead, cheeks, upper body, and extremities, typically appearing as erythematous, scaly lesions [2]. Prolonged and extensive exposure to UV light and potential toxins like tobacco aggravates this condition [3]. Histologically, DLE displays interface dermatitis as a typical finding, often accompanied by deposits of immunoglobulin M (IgM), Immunoglobulin G (IgG), and complements of the dermo-epidermal junction in the affected skin. The majority of the patients show positive Antinuclear antibodies (ANA) and mildly elevated Erythrocyte sedimentation rate (ESR) [3].

Classic DLE lesions appear as small plaques, papules, or reddish-purple macules with peripheral thickening. Histopathological examination and clinical features play a significant role in diagnosing DLE. The patients with DLE commonly undergo laboratory investigations and maintain records of ESR and Complete blood counts carried out at regular intervals. In some cases, patients with DLE might develop Systemic Lupus Erythematosus (SLE), exhibiting features synchronous to DLE. The usual treatments for DLE include topical Corticosteroids, oral anti-malarial drugs, and sunscreens for photoprotection. In refractory cases, further treatment options like thalidomide, dapsone, or retinoids are considered [4].

## Case presentation

**Figure 1:**
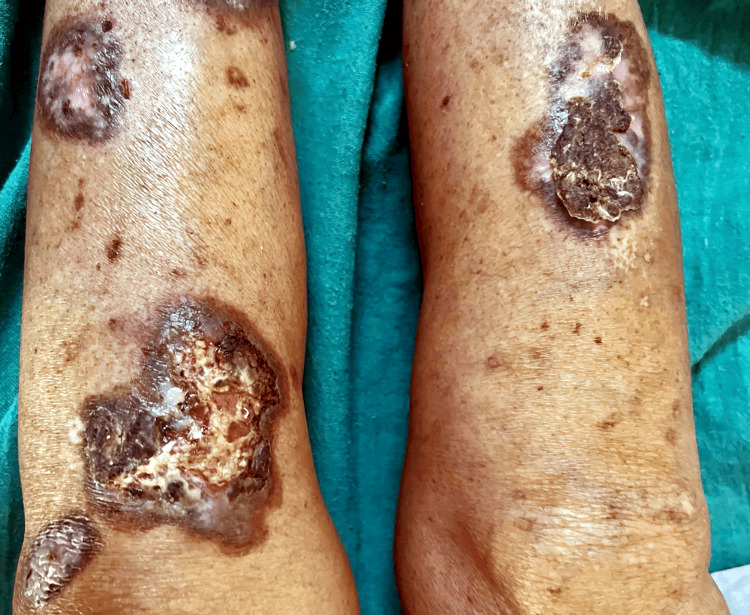
Multiple well-defined non-healing ulcers with pus oozing and crusting of various sizes present over bilateral lower limb at the time of admission.

A known hypertensive female on regular medication, in her mid-60s with no other co-morbidities presented to the Dermatology Outpatient Department complaining of painful ulcerative nonhealing lesions over her upper limb, lower limb for two months and joint pain with stiffness since the past one month. Additionally, she gave a history of seeking previous treatments from a homeopathic practitioner in the past three months with no available documentation. She also gave a history of applying Neem leaf paste over the lesions for the past month, which further led to the aggravation of the lesions. Multiple well-defined non-healing ulcers with crusting of various sizes present over bilateral upper and lower limbs ranging from 3 x 3 cm and 5 x 5 cm (Figure 1).

**Figure 2:**
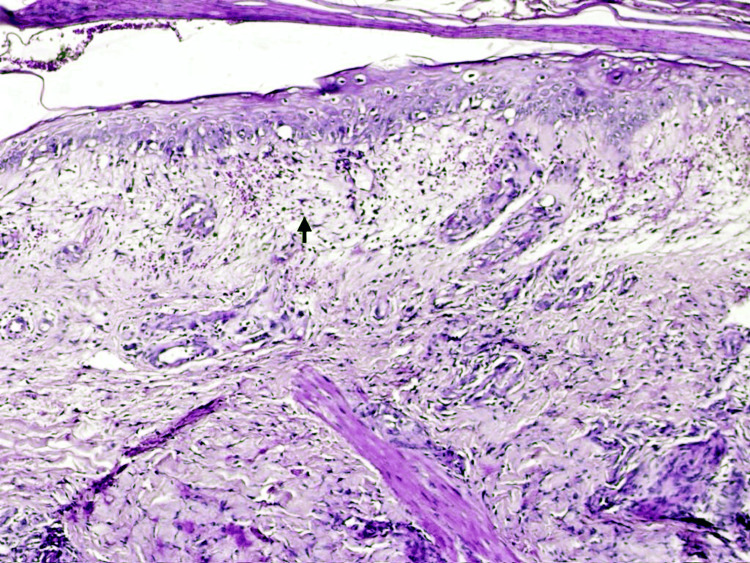
H&E stain of a biopsy taken from the perilesional site at the time of admission showing upper and papillary dermis showing intense inflammation and severe edema mixed with lymphocyte and neutrophilic infiltrate (black arrow).

On admission to the ward, a routine workup was done. Furthermore, a pus culture sent from the oozing wounds turned out positive for Staphylococcus growth and the punch biopsy taken from the edge of the ulcer margin of the lower limb which on histopathology showed neutrophilic infiltration (Figure 2). The management consisted of saline and potassium permanganate compress over the lesions, alongside intravenous, oral, and topical antibiotics. The patient was started on oral steroids tablet prednisolone 0.75 mg per kilogram twice a day, intravenous antibiotics Injection Amoxicillin, and Potassium Clavulanate 1.2 g twice a day for a week. Adequate wound care was administered, involving drainage of pus-filled lesions followed by topical antibiotic creams and adjustments in antibiotic therapy based on sensitivity patterns.

**Figure 3:**
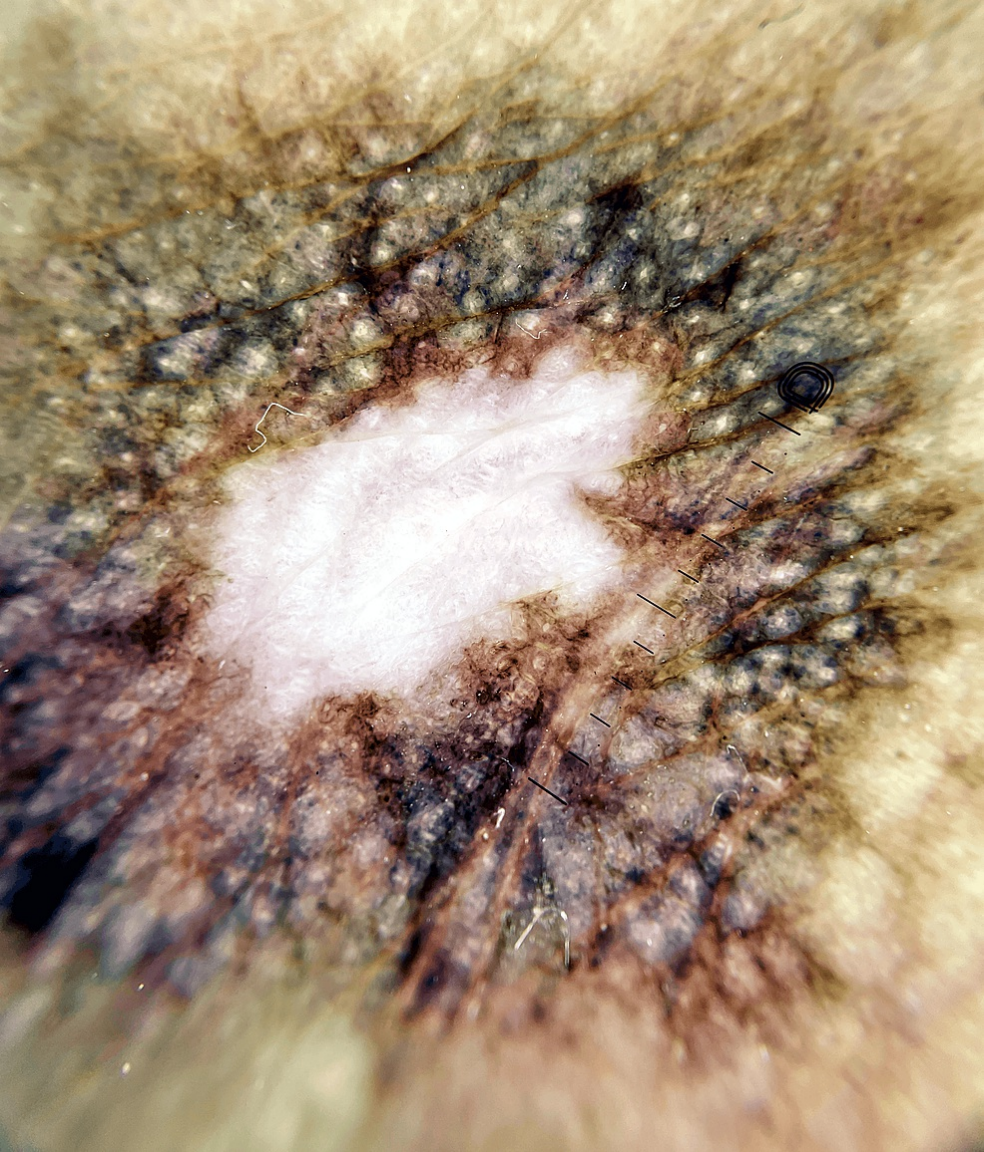
Dermoscopic image taken from the healed patch over right thigh showing central depigmentation surrounded by a peripheral brown hyperpigmented rim with the absence of follicular opening.

As the wounds healed, further investigations were advised, and an Anti-Nuclear Antibody (ANA) was sent. ANA revealed a positive 1:80 titer exhibiting a Speckled pattern on Immunofluorescence, suggesting the presence of a connective tissue disorder. Dermoscopic examination taken from a healing lower limb ulcer unveiled characteristic “Central depigmentation surrounded by a peripheral brown hyperpigmented rim with the absence of follicular opening” appearance (Figure 3). Therefore, an oral antimalarial tablet Hydroxycloroquine 200 mg once a day was added to the treatment plan in order to further taper down oral steroids.

**Figure 4:**
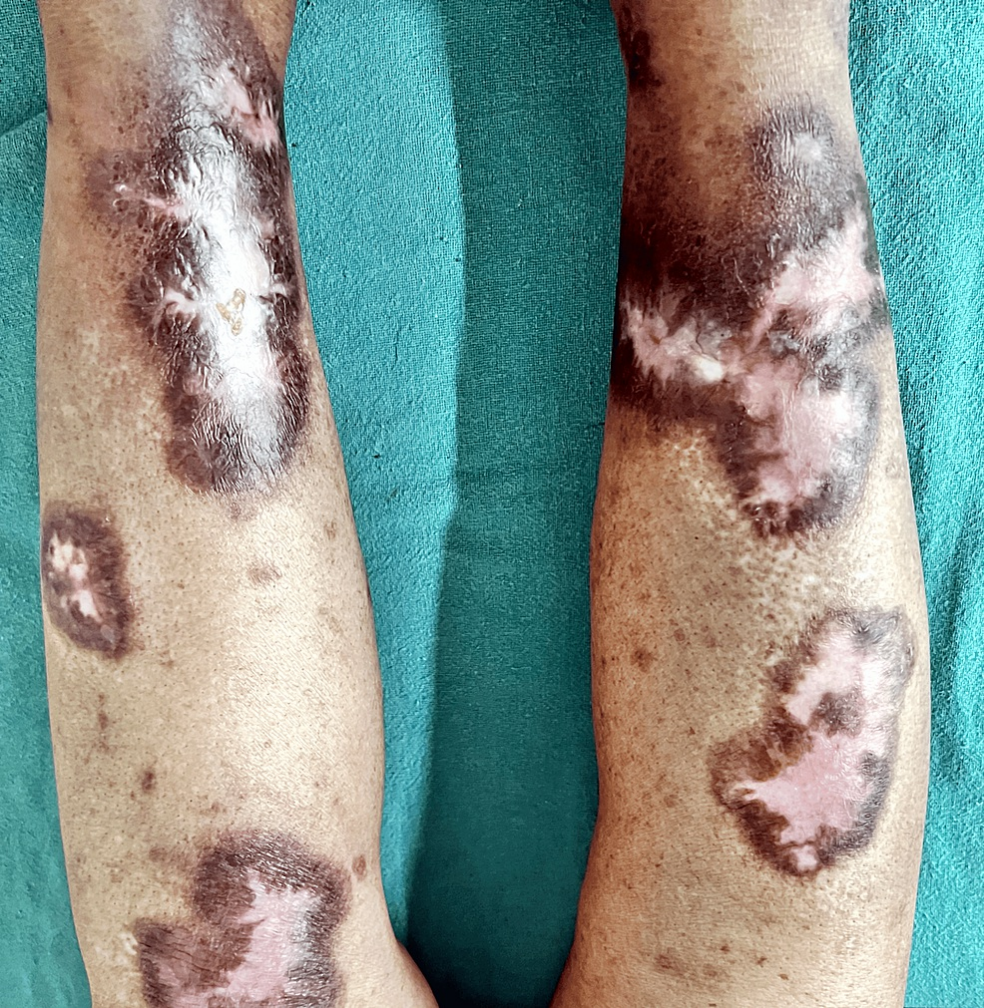
Multiple well-defined healed hyperpigmented patches with central depigmentation of various sizes present over bilateral lower limb (after three weeks of treatment).

After three weeks of indoor treatment, ulcers healed with central depigmented and surrounding hyperpigmentation (Figure 4).

The combination of history, clinical examination, dermoscopy findings, histopathological, and ANA serology collectively led to a conclusive diagnosis of ulcerative DLE. Hence, such critical assessments and diagnostic tools provided a comprehensive understanding in confirming the specific subtype of Cutaneous Lupus Erythematosus.

Presently, the patient continues to seek care through regular monthly follow-ups in the Dermatology OPD with ongoing management consisting of a tablet of Hydroxychloroquine 200 mg once a day, a tablet of Methotrexate 5 mg once a week, and a tablet of Folic acid daily once a day, oral antihistamines tablet Levocetirizine Dihydrochloride 5 mg taken before bedtime and the local application of Emollients over the resolving central depigmented and surrounded hyperpigmented patches.

## Discussion

The case holds a distinctive value, as the presenting features of the patient were corresponding with the clinical presentation of pyoderma gangrenosum. Pyoderma gangrenosum is a rare ulcerative disease. It is a neutrophilic dermatosis with a healing pattern showing a “Cribriform (Cigarette paper) scar” [5]. Felipe Soto et al in their article on pyoderma gangrenosum, described the features and treatment which coincide with the clinical features and step-by-step management of a discoid lupus case. They emphasized ruling out connective tissue disorders before confirming the diagnosis of pyoderma gangrenosum as both pyoderma and connective tissue disorder mimic each other in the initial stage of presentations [6]. Usually, a case of pyoderma gangrenosum shows signs of healing after a complete course of Injectable, oral, and topical antibiotics and steroids [5]. In this patient, the healing stage was late to set in and required careful changes in the antibiotic regimen as per the pus culture sensitivity report along with wound debridement, and other anti-septic precautions. As the wounds healed, the clinical presentation did not align with the healing clinical pattern of pyoderma gangrenosum. Salah et al. [7] describe chronic cutaneous lupus erythematosus usually present with sparing of head and neck area. Most of the lesions on the limbs and back were resolved with central depigmentation and a brown rim of hyperpigmentation surrounding the area with white flaking of skin and arborizing vessels. David-Bajar et al. [3] discussed immunofluorescence patterns to differentiate the variant cases of lupus erythematosus. In our case, we found the ANA titer weakly positive, confirming the diagnosis.

Antimalarials are considered first-line treatment, tablet Hydroxychloroquine (HCQ) is started at the dose of 200 mg once a day after ophthalmology clearance and is gradually increased to 200 mg twice a day [8]. Another drug used in the management of this condition is Azathioprine, which is a purine synthesis inhibitor with immunosuppressive effects, Thiopurine methyltransferase test (TPMT) measurement is a must before starting the drug. Systemic retinoids can be considered for modifying the function of epidermal keratinocytes. A prior liver profile analysis is required before adding the drug to the regimen [9]. Methotrexate is a second-line drug, that acts rapidly to help control the disease progression, thus gradually withdrawn when alternative drugs show their action. Topical sun protection, topical corticosteroids, and measures such as smoking cessation as well as other symptomatic measures are to be taken to fasten the healing period [10]. Thus, looking at the severity of the lesions and post-treatment scenario, the case holds a high value leading to the importance of clinical diagnosis, dermoscopy, and immunofluorescence [1]. 

## Conclusions

Ulcerative DLE, a manifestation of chronic cutaneous lupus erythematosus without involvement of the head and neck region, emerges as a distinctive rarity in dermatological diagnoses. Its resemblance to pyoderma gangrenosum often leads to confusion during initial assessments. However, the tell-tale signs of Ulcerative DLE reveal themselves during the healing phase, a distinct pattern characterized by central scarring encircled by a pigmented rim, offering a crucial diagnostic clue. Notably, cutaneous lupus erythematosus may not always present with the textbook classical features, adding a layer of complexity to its identification. This subtlety necessitates serial follow-ups, each visit revealing incremental insights crucial for a retrospective diagnosis. The evolving nature of these dermatological conditions underscores the importance of maintaining a readiness to re-evaluate and revise initial diagnostic impressions. Instances, where conditions like pemphigus or pyoderma gangrenosum overlap with lupus, emphasize the necessity for vigilant monitoring and a flexible diagnostic approach, ensuring accurate identification and tailored management for optimal patient care thus guiding us toward sharper diagnoses and more nuanced treatment strategies.
